# Crystal structure and Hirshfeld surface analysis of 3-(4-meth­oxy­phen­yl)-1-methyl-4-phenyl-1*H*-pyrazolo­[3,4-*d*]pyrimidine

**DOI:** 10.1107/S2056989019004894

**Published:** 2019-04-16

**Authors:** Mohamed El Hafi, Sevgi Kansiz, Sanae Lahmidi, Mohammed Boulhaoua, Youssef Ramli, Necmi Dege, El Mokhtar Essassi, Joel T. Mague

**Affiliations:** a Laboratoire de Chimie Organique Hétérocyclique, Centre de Recherche Des Sciences des Médicaments, Pôle de Compétence Pharmacochimie, Av Ibn Battouta, BP 1014, Faculté des Sciences, Université Mohammed V, Rabat, Morocco; b Ondokuz Mayıs University, Faculty of Arts and Sciences, Department of Physics, 55139, Kurupelit, Samsun, Turkey; c Laboratory of Medicinal Chemistry, Faculty of Medicine and Pharmacy, Mohammed V, University Rabat, Morocco; d Department of Chemistry, Tulane University, New Orleans, LA 70118, USA

**Keywords:** crystal structure, hydrogen bond, C—H⋯π(ring) inter­actions, pyrazolo­pyrimidine, Hirshfeld surface analysis

## Abstract

In the title mol­ecule, C_19_H_16_N_4_O, the planar pyrazolo­pyrimidine moiety is inclined to the attached phenyl rings by 35.42 (4) and 54.51 (6)°. In the crystal, adjacent mol­ecules are linked into chains parallel to [110] and [10] by C—H⋯O and C—H⋯N hydrogen bonds. Additional C—H⋯π(ring) inter­actions lead to the formation of the final three-dimensional network structure. The Hirshfeld surface analysis of the title compound suggests that the most significant contributions to the crystal packing are from H⋯H (48.2%), C⋯H/H⋯C (23.9%) and N⋯H/H⋯N (17.4%) contacts.

## Abstract 2

In the crystal structure of the title compound, C—H⋯O and C—H⋯N hydrogen bonds as well as C—H⋯π(ring) inter­actions link individual mol­ecules into a three-dimensional network.

## Chemical context   

**Figure 1:**
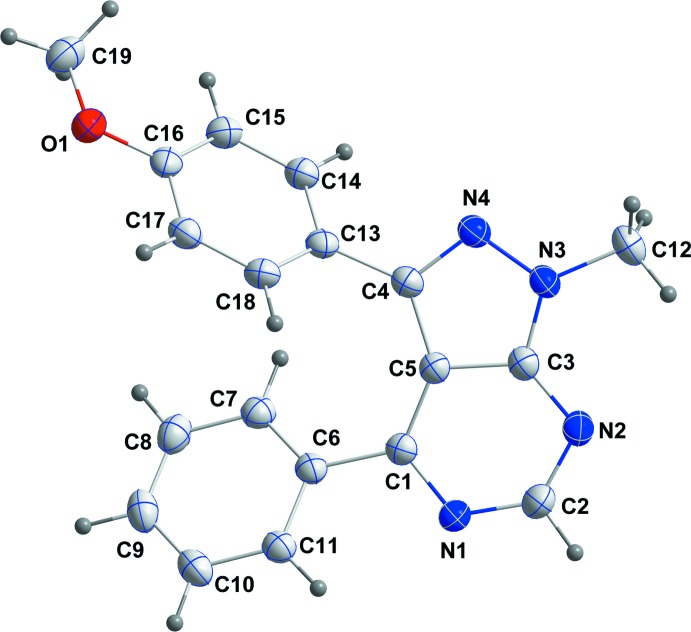
The title mol­ecule with the labeling scheme and displacement ellipsoids drawn at the 50% probability level.

Pyrazolo­[3,4-*d*]pyrimidine derivatives represent an important class of compounds because of their potent biological activities and thus find applications as anti­proliferative (Tallani *et al.*, 2010 ▸), anti­bacterial (Rostamizadeh *et al.*, 2013 ▸) or anti­tumor agents (Tintori *et al.*, 2015 ▸). The present contribution is a continuation of the investigation of pyrazolo­[3,4-*d*]pyrimi­dine derivatives recently published by us (El Hafi *et al.*, 2017 ▸, 2018*a* ▸,*b* ▸). We report herein the synthesis, mol­ecular and crystal structures of the title compound, 3-(4-meth­oxy­phen­yl)-1-methyl-4-phenyl-1*H*-pyrazolo­[3,4-*d*]pyrimidine (Fig. 1 ▸), along with the results of a Hirshfeld surface analysis.
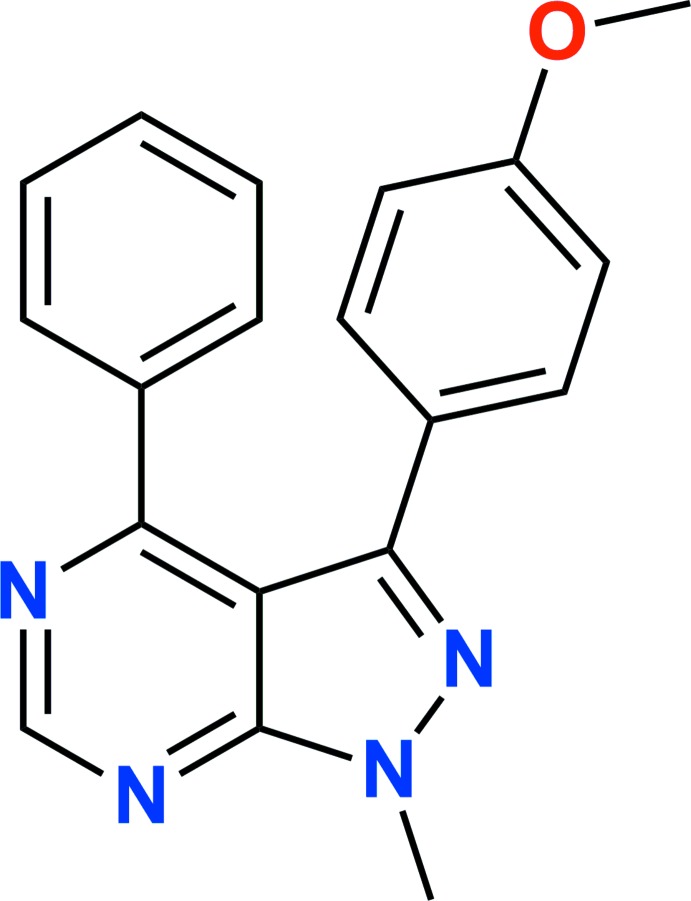


## Structural commentary   

The heterocyclic ring system is planar (r.m.s. deviation of the fitted atoms = 0.0194 Å) with a maximum displacement of 0.0329 (10) Å from the mean plane for atom C1. The attached benzene rings (C6–C11 and C13–C18) are inclined to the above plane by 35.42 (4) and 54.51 (6)°, respectively.

## Supra­molecular features   

**Figure 2:**
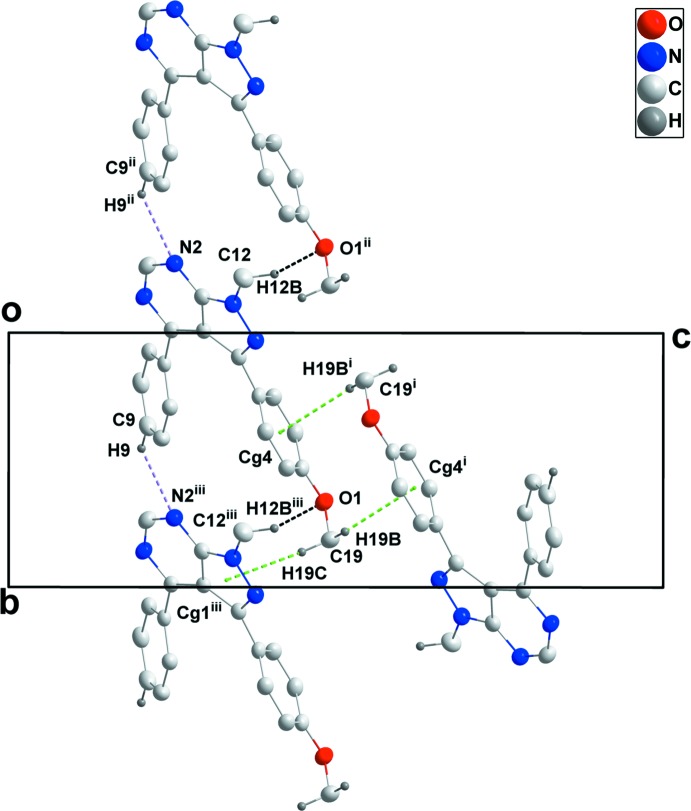
Details of the inter­molecular inter­actions in a view along [100]. C—H⋯O and C—H⋯N hydrogen bonds are shown, respectively, as black and light-purple dashed lines while the C—H⋯π(ring) inter­actions are shown as green dashed lines. [Symmetry codes: (i) −*x* + 1, −*y* + 1, −*z* + 1; (ii) *x* + 1, *y* − 1, *z*; (iii) *x* − 1, *y* + 1, *z*.]

In the crystal, a combination of C9—H9⋯N2 hydrogen bonds between aromatic hydrogen atoms and one of the pyrimidine N atoms as well as C12—H12*B*⋯O1 hydrogen bonds between a methyl H atom and the meth­oxy O atoms of adjacent mol­ecules lead to the formation of chains extending alternately parallel to [110] and [1
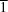
0] (Table 1 ▸ and Fig. 2 ▸). Centrosymmetric dimers with an 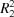
(8) graph-set motif are formed by pairwise C17—H17⋯O1 hydrogen bonds. The chains are linked into layers parallel to (001) by C19—H19*C*⋯*Cg*1 inter­actions, and pairs of layers are joined into thicker slabs by C19—H19*B*⋯*Cg*4 inter­actions (Table 1 ▸ and Figs. 2 ▸–4 ▸ ▸).

## Database survey   

A search of the Cambridge Structural Database (CSD, version 5.40, update November 2018; Groom *et al.*, 2016 ▸) for the 1-methyl-1*H*-pyrazolo­[3,4-*d*]pyrimidine skeleton yielded seven hits. In all of these structures, the pyrazolo­[3,4-*d*]pyrimi­dine rings are planar, as in the title compound. In FEWVIP (El Hafi *et al.*, 2018*a* ▸), centrosymmetric dimers with an 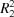
(8) graph set motif are formed by pairwise N—H⋯O hydrogen bonds; the dimers are connected into chains parallel to [001], similar to those in the title compound. Neighbouring mol­ecules in FAXFEP (Sheldrick & Bell, 1987*a* ▸) and in FOGXAA, FOGXEE, FOGXII, JAGROY (Sheldrick & Bell, 1987*b* ▸) are linked by N—H⋯O hydrogen bonds, whereas in XAXRUM (El Fal *et al.*, 2017 ▸), C—H⋯N hydrogen bonds are responsible for the formation of double chains running parallel to [100].

## Hirshfeld surface analysis   

**Figure 5:**
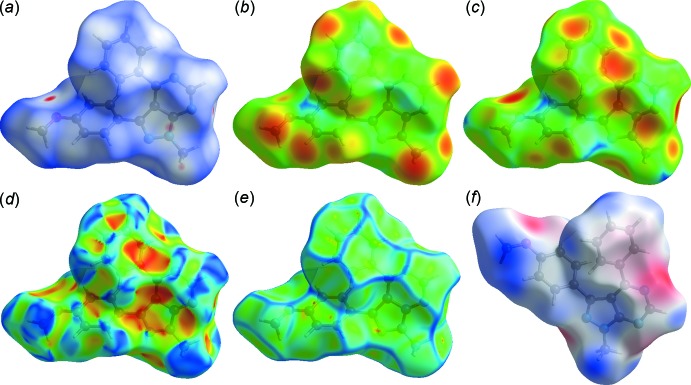
The Hirshfeld surfaces of the title compound mapped over (*a*) *d*_norm_, (*b*) *d*_i_, (*c*) *d*_e_, (*d*) shape-index, (*e*) curvedness and (*f*) electrostatic potential.

**Figure 6:**
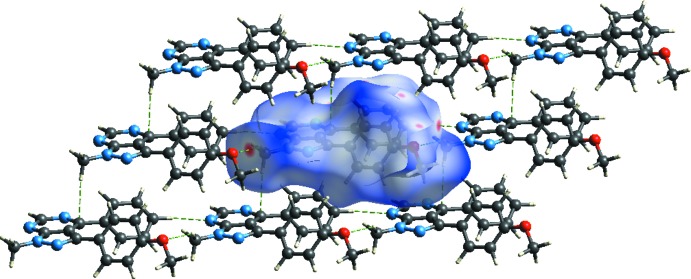
*d*_norm_ mapped on Hirshfeld surfaces for visualizing the inter­molecular inter­actions of the title compound.

*CrystalExplorer17* (Turner *et al.*, 2017 ▸) was used to perform the Hirshfeld surface analysis (Spackman & Jayatilaka, 2009 ▸) and obtain the associated two-dimensional fingerprint plots (McKinnon *et al.*, 2007 ▸). Fig. 5 ▸ shows *d_norm_*, *d_i_*, *d_e_*, shape-index, curvedness and electrostatic potential mapped over the Hirshfeld surface for the title compound while Fig. 6 ▸ illustrates the Hirshfeld surface of the mol­ecule in the crystal, with the evident hydrogen-bonding inter­actions indicated as intense red spots.

**Figure 7:**
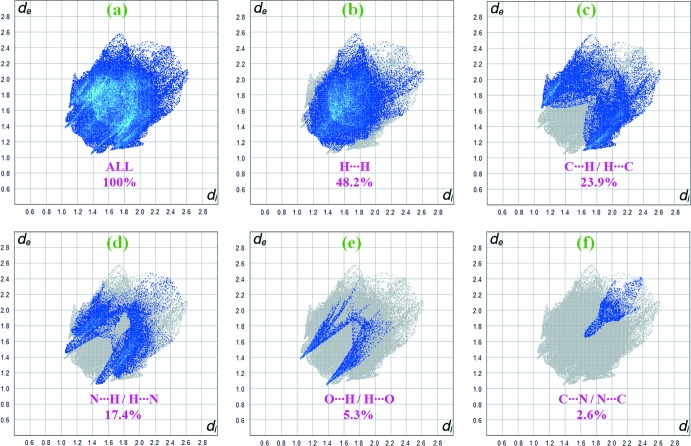
Two-dimensional fingerprint plots for the title structure, with a *d*_norm_ view and relative contribution of the atom pairs to the Hirshfeld surface.

Fig. 7 ▸ *a* shows the two-dimensional fingerprint of the sum of the contacts contributing to the Hirshfeld surface represented in normal mode. The fingerprint plots provide information about the percentage contributions of various inter­atomic contacts in the structure. The blue color refers to the frequency of occurrence of the (*d*_i_, *d*_e_) pair with the full fingerprint outlined in gray. Individual fingerprint plots (Fig. 7 ▸ *b*) reveal that the H⋯H contacts clearly give the most significant contribution to the Hirshfeld surface (48.2%). In addition, C⋯H/H⋯C, N⋯H/H⋯N, O⋯H/H⋯O and C⋯N/N⋯C contacts contribute 23.9%, 17.4%, 5.3% and 2.6%, respectively, to the Hirshfeld surface. In particular, the N⋯H/H⋯N and O⋯H/H⋯O contacts indicate the presence of inter­molecular C—H⋯N and C—H⋯O inter­actions, respectively. Much weaker C⋯C (2.2%) and C⋯O/O⋯C (0.5%) contacts also occur.

**Figure 8:**
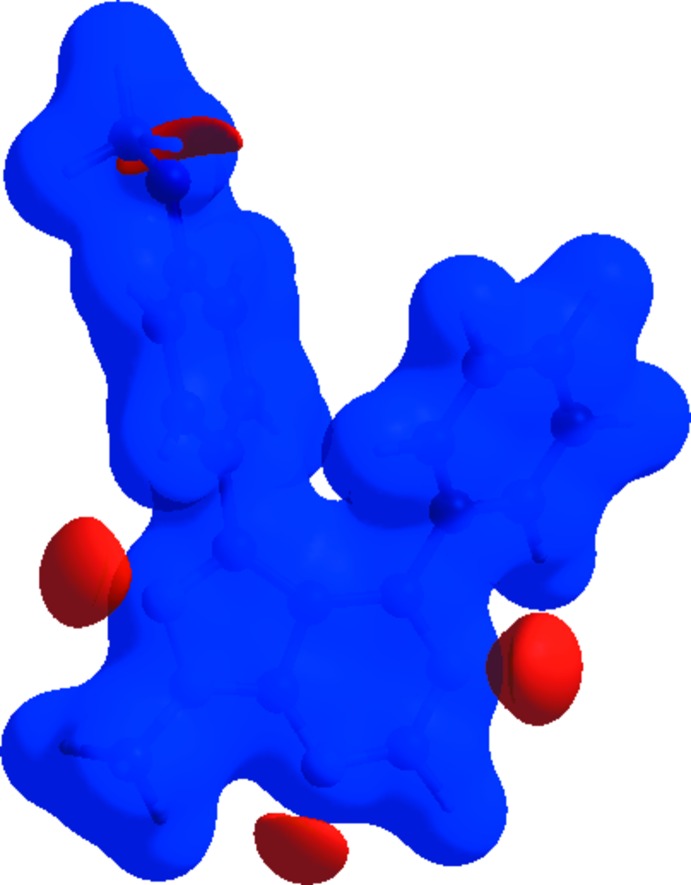
A view of the mol­ecular electrostatic potential of the title compound in the range −0.05 to 0.05 a.u. using the 6–31G(d,p) basis set (DFT method).

A view of the mol­ecular electrostatic potential, in the range −0.0500 to 0.0500 a.u. using the 6-31G(d,p) basis set (DFT method), for the title compound is shown in Fig. 8 ▸. The donors and acceptors for C—H⋯O and C—H⋯N hydrogen bonds are shown as blue and red areas around the atoms related with positive (hydrogen-bond donors) and negative (hydrogen-bond acceptors) electrostatic potentials, respectively.

## Synthesis and crystallization   

Under an atmosphere of argon, a mixture of 1-methyl-4-phenyl-1*H*-pyrazolo­[3,4-*d*]pyrimidine (0.1 g, 0.47 mmol), 4-iodo­anisole (0.22 g, 0.95 mmol), Cs_2_CO_3_ (0.46g, 1.42 mmol), K_3_PO_4_ (0.25 g, 1.18 mmol), 1,10-phenanthroline (0.034 g, 0.19 mmol), and Pd(OAc)_2_ (0.021 g, 0.094 mmol) in DMA (3 ml) was heated to 438 K for 48 h. After completion of the reaction, the mixture was allowed to cool to room temperature and the solvent was removed under reduced pressure. Water (15 ml) was added, and the resulting aqueous phase was extracted with CH_2_Cl_2_ (3 × 15 ml). The combined organic layers were dried with MgSO_4_ and concentrated under vacuum. The residue was purified by column chromatography on silica gel (EtOAc/petroleum ether). The title compound was recrystallized from ethanol at room temperature, giving colorless crystals (yield: 71%; m.p. 412–414 K).

## Refinement   

Crystal data, data collection and structure refinement details are summarized in Table 2 ▸. All H atoms were located in a difference-Fourier map and were freely refined.

## Supplementary Material

Crystal structure: contains datablock(s) global, I. DOI: 10.1107/S2056989019004894/wm5499sup1.cif

Structure factors: contains datablock(s) I. DOI: 10.1107/S2056989019004894/wm5499Isup2.hkl

Supporting information file. DOI: 10.1107/S2056989019004894/wm5499Isup3.cml

CCDC reference: 1909062

Additional supporting information:  crystallographic information; 3D view; checkCIF report

## Figures and Tables

**Figure 3:**
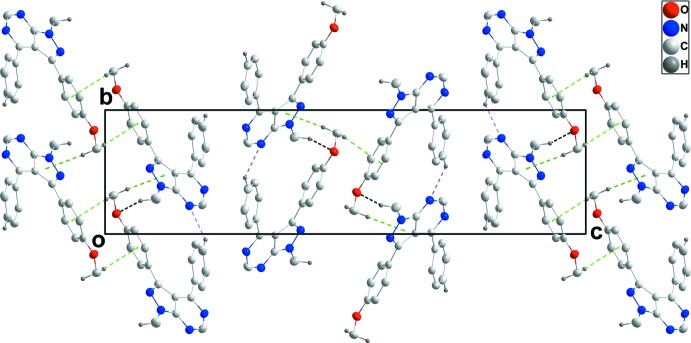
Packing of the crystal viewed along [100] with inter­molecular inter­actions depicted as in Fig. 2 ▸.

**Figure 4:**
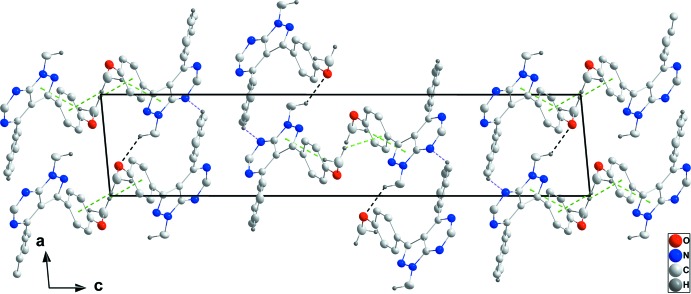
Packing of the crystal viewed along [010] with inter­molecular inter­actions depicted as in Fig. 2 ▸.

**Table 1:** Hydrogen-bond geometry (Å, °) *Cg*1 and *Cg*4 are the centroids of the C3/C4/C5/N4/N3 and C13–C18 rings, respectively.

*D*—H⋯*A*	*D*—H	H⋯*A*	*D*⋯*A*	*D*—H⋯*A*
C9—H9⋯N2^i^	0.988 (18)	2.579 (17)	3.3995 (19)	140.3 (14)
C12—H12*B*⋯O1^ii^	0.98 (2)	2.49 (2)	3.2694 (19)	136.4 (15)
C17—H17⋯O1^iii^	0.983 (17)	2.618 (9)	3.4973 (17)	149.3 (14)
C19—H19*B*⋯*Cg*4^iv^	1.02 (2)	2.74 (2)	3.5928 (19)	141.9 (14)
C19—H19*C*⋯*Cg*1^v^	0.995 (19)	2.947 (19)	3.9072 (19)	162.0 (15)

Symmetry codes: (i) 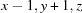; (ii) 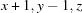; (iii) 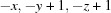; (iv) 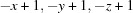; (v) 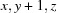.

**Table 2:** Experimental details

Crystal data	
Chemical formula	C_19_H_16_N_4_O
*M*_r_	316.36
Crystal system, space group	Monoclinic, *P*2_1_/*n*
Temperature (K)	150
*a*, *b*, *c* (Å)	6.5227 (3), 7.8979 (4), 30.7774 (15)
β (°)	95.389 (2)
*V* (Å^3^)	1578.51 (13)
*Z*	4
Radiation type	Cu *K*α
μ (mm^−1^)	0.69
Crystal size (mm)	0.30 × 0.24 × 0.04

Data collection	
Diffractometer	Bruker D8 VENTURE PHOTON 100 CMOS
Absorption correction	Multi-scan (*SADABS*; Krause *et al.*, 2015 ▸)
*T*_min_, *T*_max_	0.85, 0.98
No. of measured, independent and observed [*I* > 2σ(*I*)] reflections	11542, 3069, 2613
*R*_int_	0.035
(sin θ/λ)_max_ (Å^−1^)	0.617

Refinement	
*R*[*F* ^2^ > 2σ(*F* ^2^)], *wR*(*F* ^2^), *S*	0.039, 0.091, 1.06
No. of reflections	3069
No. of parameters	282
H-atom treatment	All H-atom parameters refined
Δρ_max_, Δρ_min_ (e Å^−3^)	0.19, −0.18

Computer programs: *APEX3* and *SAINT* (Bruker, 2016 ▸), *SAINT* (Bruker, 2016 ▸), *SHELXT* (Sheldrick, 2015*a* ▸), *SHELXL2018* (Sheldrick, 2015*b* ▸), *DIAMOND* (Brandenburg & Putz, 2012 ▸) and *SHELXTL* (Sheldrick, 2008 ▸).

## supplementary crystallographic information

### Crystal data

**Table:**

C_19_H_16_N_4_O	*F*(000) = 664
*M**_r_* = 316.36	*D*_x_ = 1.331 Mg m^−^^3^
Monoclinic, *P*2_1_/*n*	Cu *K*α radiation, λ = 1.54178 Å
*a* = 6.5227 (3) Å	Cell parameters from 8514 reflections
*b* = 7.8979 (4) Å	θ = 2.9–72.2°
*c* = 30.7774 (15) Å	µ = 0.69 mm^−^^1^
β = 95.389 (2)°	*T* = 150 K
*V* = 1578.51 (13) Å^3^	Plate, colourless
*Z* = 4	0.30 × 0.24 × 0.04 mm

### Data collection

**Table:**

Bruker D8 VENTURE PHOTON 100 CMOS diffractometer	3069 independent reflections
Radiation source: INCOATEC IµS micro-focus source	2613 reflections with *I* > 2σ(*I*)
Mirror monochromator	*R*_int_ = 0.035
Detector resolution: 10.4167 pixels mm^-1^	θ_max_ = 72.2°, θ_min_ = 5.8°
ω scans	*h* = −7→8
Absorption correction: multi-scan (*SADABS*; Krause *et al.*, 2015)	*k* = −9→8
*T*_min_ = 0.85, *T*_max_ = 0.98	*l* = −37→33
11542 measured reflections	

### Refinement

**Table:**

Refinement on *F*^2^	Hydrogen site location: difference Fourier map
Least-squares matrix: full	All H-atom parameters refined
*R*[*F*^2^ > 2σ(*F*^2^)] = 0.039	*w* = 1/[σ^2^(*F*_o_^2^) + (0.0337*P*)^2^ + 0.5793*P*] where *P* = (*F*_o_^2^ + 2*F*_c_^2^)/3
*wR*(*F*^2^) = 0.091	(Δ/σ)_max_ < 0.001
*S* = 1.06	Δρ_max_ = 0.19 e Å^−^^3^
3069 reflections	Δρ_min_ = −0.18 e Å^−^^3^
282 parameters	Extinction correction: *SHELXL2018* (Sheldrick, 2015*b*), Fc^*^=kFc[1+0.001xFc^2^λ^3^/sin(2θ)]^-1/4^
0 restraints	Extinction coefficient: 0.0035 (3)

### Special details

**Table:**

Geometry. All esds (except the esd in the dihedral angle between two l.s. planes) are estimated using the full covariance matrix. The cell esds are taken into account individually in the estimation of esds in distances, angles and torsion angles; correlations between esds in cell parameters are only used when they are defined by crystal symmetry. An approximate (isotropic) treatment of cell esds is used for estimating esds involving l.s. planes.
Refinement. Refinement of F^2^ against ALL reflections. The weighted R-factor wR and goodness of fit S are based on F^2^, conventional R-factors R are based on F, with F set to zero for negative F^2^. The threshold expression of F^2^ > 2sigma(F^2^) is used only for calculating R-factors(gt) etc. and is not relevant to the choice of reflections for refinement. R-factors based on F^2^ are statistically about twice as large as those based on F, and R- factors based on ALL data will be even larger.

### Fractional atomic coordinates and isotropic or equivalent isotropic displacement parameters (Å^2^)

**Table:**

	*x*	*y*	*z*	*U*_iso_*/*U*_eq_	
O1	0.20599 (16)	0.66433 (13)	0.47647 (3)	0.0355 (3)	
N1	0.21981 (18)	−0.14300 (15)	0.29349 (4)	0.0310 (3)	
N2	0.53402 (18)	−0.27278 (15)	0.32443 (4)	0.0314 (3)	
N3	0.70112 (17)	−0.11435 (15)	0.38386 (4)	0.0302 (3)	
N4	0.66731 (18)	0.02986 (15)	0.40680 (4)	0.0306 (3)	
C1	0.2383 (2)	−0.01161 (17)	0.32134 (4)	0.0257 (3)	
C2	0.3656 (2)	−0.26430 (19)	0.29691 (5)	0.0338 (3)	
H2	0.343 (3)	−0.360 (2)	0.2760 (6)	0.038 (4)*	
C3	0.5468 (2)	−0.14182 (18)	0.35243 (4)	0.0271 (3)	
C4	0.4895 (2)	0.09486 (18)	0.38968 (4)	0.0267 (3)	
C5	0.4044 (2)	−0.00839 (17)	0.35407 (4)	0.0249 (3)	
C6	0.0795 (2)	0.12152 (17)	0.31411 (4)	0.0257 (3)	
C7	0.1276 (2)	0.29264 (18)	0.31985 (5)	0.0305 (3)	
H7	0.270 (3)	0.330 (2)	0.3290 (5)	0.034 (4)*	
C8	−0.0249 (3)	0.4138 (2)	0.31144 (5)	0.0366 (4)	
H8	0.017 (3)	0.533 (2)	0.3158 (6)	0.042 (5)*	
C9	−0.2243 (2)	0.3662 (2)	0.29735 (5)	0.0379 (4)	
H9	−0.331 (3)	0.454 (2)	0.2911 (6)	0.044 (5)*	
C10	−0.2719 (2)	0.1971 (2)	0.29048 (5)	0.0347 (3)	
H10	−0.408 (3)	0.162 (2)	0.2792 (6)	0.051 (5)*	
C11	−0.1211 (2)	0.07493 (19)	0.29875 (5)	0.0287 (3)	
H11	−0.153 (2)	−0.048 (2)	0.2930 (5)	0.033 (4)*	
C12	0.8835 (2)	−0.2161 (2)	0.39446 (6)	0.0370 (4)	
H12A	1.000 (3)	−0.162 (3)	0.3834 (7)	0.062 (6)*	
H12B	0.912 (3)	−0.232 (2)	0.4259 (7)	0.057 (6)*	
H12C	0.855 (3)	−0.330 (3)	0.3805 (6)	0.054 (5)*	
C13	0.4119 (2)	0.24828 (17)	0.41004 (4)	0.0267 (3)	
C14	0.5384 (2)	0.38855 (18)	0.41690 (5)	0.0298 (3)	
H14	0.678 (3)	0.386 (2)	0.4064 (5)	0.037 (4)*	
C15	0.4749 (2)	0.53140 (18)	0.43856 (5)	0.0311 (3)	
H15	0.565 (2)	0.6368 (18)	0.4445 (5)	0.021 (3)*	
C16	0.2816 (2)	0.53215 (18)	0.45402 (4)	0.0291 (3)	
C17	0.1517 (2)	0.39289 (19)	0.44698 (5)	0.0313 (3)	
H17	0.017 (3)	0.394 (2)	0.4587 (6)	0.041 (5)*	
C18	0.2157 (2)	0.25287 (19)	0.42500 (5)	0.0300 (3)	
H18	0.124 (2)	0.157 (2)	0.4201 (5)	0.033 (4)*	
C19	0.3310 (3)	0.8127 (2)	0.48265 (6)	0.0407 (4)	
H19A	0.252 (3)	0.891 (3)	0.5003 (6)	0.057 (6)*	
H19B	0.471 (3)	0.782 (2)	0.4976 (7)	0.054 (5)*	
H19C	0.356 (3)	0.862 (2)	0.4538 (6)	0.046 (5)*	

### Atomic displacement parameters (Å^2^)

**Table:**

	*U*^11^	*U*^22^	*U*^33^	*U*^12^	*U*^13^	*U*^23^
O1	0.0375 (6)	0.0338 (6)	0.0346 (6)	0.0032 (4)	0.0009 (4)	−0.0060 (4)
N1	0.0310 (6)	0.0318 (7)	0.0298 (6)	0.0027 (5)	0.0009 (5)	−0.0055 (5)
N2	0.0314 (6)	0.0314 (6)	0.0314 (7)	0.0052 (5)	0.0035 (5)	−0.0016 (5)
N3	0.0262 (6)	0.0331 (7)	0.0304 (6)	0.0050 (5)	−0.0010 (5)	0.0001 (5)
N4	0.0277 (6)	0.0338 (7)	0.0297 (6)	0.0020 (5)	0.0001 (5)	−0.0002 (5)
C1	0.0248 (6)	0.0284 (7)	0.0240 (7)	−0.0009 (5)	0.0034 (5)	0.0004 (5)
C2	0.0348 (8)	0.0334 (8)	0.0330 (8)	0.0031 (6)	0.0027 (6)	−0.0068 (6)
C3	0.0261 (7)	0.0297 (7)	0.0258 (7)	0.0013 (5)	0.0039 (5)	0.0019 (6)
C4	0.0251 (6)	0.0295 (7)	0.0254 (7)	−0.0005 (5)	0.0012 (5)	0.0013 (5)
C5	0.0247 (6)	0.0265 (7)	0.0238 (7)	0.0006 (5)	0.0031 (5)	0.0008 (5)
C6	0.0272 (7)	0.0298 (7)	0.0201 (6)	0.0023 (5)	0.0022 (5)	0.0008 (5)
C7	0.0340 (8)	0.0304 (7)	0.0265 (7)	0.0003 (6)	−0.0006 (6)	0.0004 (6)
C8	0.0489 (9)	0.0295 (8)	0.0312 (8)	0.0066 (7)	0.0029 (7)	0.0009 (6)
C9	0.0402 (8)	0.0421 (9)	0.0320 (8)	0.0161 (7)	0.0060 (6)	0.0047 (7)
C10	0.0274 (7)	0.0465 (9)	0.0302 (8)	0.0059 (6)	0.0024 (6)	0.0043 (7)
C11	0.0277 (7)	0.0333 (8)	0.0251 (7)	0.0008 (6)	0.0025 (5)	0.0006 (6)
C12	0.0273 (8)	0.0407 (9)	0.0421 (10)	0.0082 (6)	−0.0004 (7)	0.0055 (7)
C13	0.0274 (7)	0.0304 (7)	0.0216 (7)	0.0007 (5)	−0.0017 (5)	−0.0004 (5)
C14	0.0268 (7)	0.0338 (8)	0.0285 (7)	−0.0015 (6)	0.0009 (6)	−0.0003 (6)
C15	0.0322 (7)	0.0304 (8)	0.0301 (8)	−0.0036 (6)	0.0001 (6)	−0.0010 (6)
C16	0.0330 (7)	0.0312 (7)	0.0219 (7)	0.0049 (6)	−0.0030 (5)	−0.0006 (5)
C17	0.0252 (7)	0.0395 (8)	0.0286 (7)	0.0013 (6)	0.0001 (6)	−0.0010 (6)
C18	0.0270 (7)	0.0330 (8)	0.0294 (7)	−0.0034 (6)	−0.0010 (6)	−0.0015 (6)
C19	0.0536 (10)	0.0306 (8)	0.0374 (9)	−0.0015 (7)	0.0012 (8)	−0.0042 (7)

### Geometric parameters (Å, º)

**Table:**

O1—C16	1.3690 (17)	C9—C10	1.383 (2)
O1—C19	1.430 (2)	C9—H9	0.988 (18)
N1—C1	1.3441 (18)	C10—C11	1.385 (2)
N1—C2	1.3471 (18)	C10—H10	0.96 (2)
N2—C2	1.3243 (19)	C11—H11	1.003 (16)
N2—C3	1.3438 (18)	C12—H12A	0.96 (2)
N3—C3	1.3464 (18)	C12—H12B	0.98 (2)
N3—N4	1.3687 (17)	C12—H12C	1.01 (2)
N3—C12	1.4476 (18)	C13—C14	1.3854 (19)
N4—C4	1.3310 (17)	C13—C18	1.401 (2)
C1—C5	1.4087 (19)	C14—C15	1.393 (2)
C1—C6	1.4778 (18)	C14—H14	0.997 (17)
C2—H2	0.993 (17)	C15—C16	1.389 (2)
C3—C5	1.4089 (18)	C15—H15	1.025 (15)
C4—C5	1.4351 (19)	C16—C17	1.393 (2)
C4—C13	1.4753 (19)	C17—C18	1.381 (2)
C6—C7	1.395 (2)	C17—H17	0.983 (17)
C6—C11	1.3982 (19)	C18—H18	0.973 (17)
C7—C8	1.387 (2)	C19—H19A	1.00 (2)
C7—H7	0.987 (17)	C19—H19B	1.02 (2)
C8—C9	1.384 (2)	C19—H19C	0.995 (19)
C8—H8	0.990 (18)		
			
C16—O1—C19	117.64 (12)	C9—C10—H10	121.0 (11)
C1—N1—C2	118.61 (12)	C11—C10—H10	119.0 (11)
C2—N2—C3	111.67 (12)	C10—C11—C6	120.35 (14)
C3—N3—N4	111.03 (11)	C10—C11—H11	120.7 (9)
C3—N3—C12	127.98 (13)	C6—C11—H11	118.9 (9)
N4—N3—C12	120.99 (12)	N3—C12—H12A	109.5 (12)
C4—N4—N3	107.06 (11)	N3—C12—H12B	111.7 (12)
N1—C1—C5	119.14 (12)	H12A—C12—H12B	108.8 (17)
N1—C1—C6	115.69 (12)	N3—C12—H12C	106.5 (11)
C5—C1—C6	125.16 (12)	H12A—C12—H12C	111.6 (16)
N2—C2—N1	128.53 (14)	H12B—C12—H12C	108.8 (16)
N2—C2—H2	116.0 (10)	C14—C13—C18	118.58 (13)
N1—C2—H2	115.4 (10)	C14—C13—C4	119.90 (12)
N2—C3—N3	125.60 (12)	C18—C13—C4	121.42 (12)
N2—C3—C5	126.66 (13)	C13—C14—C15	121.41 (13)
N3—C3—C5	107.73 (12)	C13—C14—H14	119.1 (10)
N4—C4—C5	110.07 (12)	C15—C14—H14	119.5 (10)
N4—C4—C13	118.00 (12)	C16—C15—C14	119.18 (13)
C5—C4—C13	131.88 (12)	C16—C15—H15	117.3 (8)
C1—C5—C3	115.24 (12)	C14—C15—H15	123.6 (8)
C1—C5—C4	140.59 (13)	O1—C16—C15	123.91 (13)
C3—C5—C4	104.08 (12)	O1—C16—C17	115.98 (13)
C7—C6—C11	119.32 (13)	C15—C16—C17	120.11 (13)
C7—C6—C1	121.63 (12)	C18—C17—C16	120.07 (13)
C11—C6—C1	118.95 (13)	C18—C17—H17	120.7 (10)
C8—C7—C6	119.76 (14)	C16—C17—H17	119.2 (10)
C8—C7—H7	119.1 (10)	C17—C18—C13	120.62 (13)
C6—C7—H7	121.1 (9)	C17—C18—H18	119.4 (9)
C9—C8—C7	120.50 (15)	C13—C18—H18	120.0 (9)
C9—C8—H8	122.8 (10)	O1—C19—H19A	105.4 (11)
C7—C8—H8	116.7 (10)	O1—C19—H19B	110.0 (11)
C10—C9—C8	120.07 (14)	H19A—C19—H19B	113.1 (16)
C10—C9—H9	120.4 (10)	O1—C19—H19C	109.8 (11)
C8—C9—H9	119.5 (10)	H19A—C19—H19C	112.3 (15)
C9—C10—C11	119.94 (14)	H19B—C19—H19C	106.3 (15)
			
C3—N3—N4—C4	−0.08 (15)	C5—C1—C6—C7	36.6 (2)
C12—N3—N4—C4	−179.99 (13)	N1—C1—C6—C11	34.01 (18)
C2—N1—C1—C5	−2.4 (2)	C5—C1—C6—C11	−147.05 (14)
C2—N1—C1—C6	176.60 (13)	C11—C6—C7—C8	1.8 (2)
C3—N2—C2—N1	2.3 (2)	C1—C6—C7—C8	178.07 (13)
C1—N1—C2—N2	−1.2 (2)	C6—C7—C8—C9	−0.1 (2)
C2—N2—C3—N3	−179.20 (14)	C7—C8—C9—C10	−1.6 (2)
C2—N2—C3—C5	0.1 (2)	C8—C9—C10—C11	1.6 (2)
N4—N3—C3—N2	−179.83 (13)	C9—C10—C11—C6	0.1 (2)
C12—N3—C3—N2	0.1 (2)	C7—C6—C11—C10	−1.8 (2)
N4—N3—C3—C5	0.79 (16)	C1—C6—C11—C10	−178.18 (13)
C12—N3—C3—C5	−179.31 (14)	N4—C4—C13—C14	52.12 (18)
N3—N4—C4—C5	−0.66 (15)	C5—C4—C13—C14	−130.58 (16)
N3—N4—C4—C13	177.20 (11)	N4—C4—C13—C18	−124.27 (15)
N1—C1—C5—C3	4.26 (19)	C5—C4—C13—C18	53.0 (2)
C6—C1—C5—C3	−174.66 (12)	C18—C13—C14—C15	0.7 (2)
N1—C1—C5—C4	−179.91 (16)	C4—C13—C14—C15	−175.81 (13)
C6—C1—C5—C4	1.2 (3)	C13—C14—C15—C16	0.7 (2)
N2—C3—C5—C1	−3.2 (2)	C19—O1—C16—C15	2.7 (2)
N3—C3—C5—C1	176.16 (12)	C19—O1—C16—C17	−177.26 (13)
N2—C3—C5—C4	179.52 (13)	C14—C15—C16—O1	178.62 (13)
N3—C3—C5—C4	−1.11 (15)	C14—C15—C16—C17	−1.4 (2)
N4—C4—C5—C1	−175.01 (16)	O1—C16—C17—C18	−179.36 (13)
C13—C4—C5—C1	7.5 (3)	C15—C16—C17—C18	0.7 (2)
N4—C4—C5—C3	1.10 (15)	C16—C17—C18—C13	0.8 (2)
C13—C4—C5—C3	−176.36 (14)	C14—C13—C18—C17	−1.4 (2)
N1—C1—C6—C7	−142.30 (14)	C4—C13—C18—C17	175.00 (13)

### Hydrogen-bond geometry (Å, º)

*Cg*1 and *Cg*4 are the centroids of the C3/C4/C5/N4/N3 and C13–C18 rings, respectively.

**Table:**

*D*—H···*A*	*D*—H	H···*A*	*D*···*A*	*D*—H···*A*
C9—H9···N2^i^	0.988 (18)	2.579 (17)	3.3995 (19)	140.3 (14)
C12—H12*B*···O1^ii^	0.98 (2)	2.49 (2)	3.2694 (19)	136.4 (15)
C17—H17···O1^iii^	0.983 (17)	2.618 (9)	3.4973 (17)	149.3 (14)
C19—H19*B*···*Cg*4^iv^	1.02 (2)	2.74 (2)	3.5928 (19)	141.9 (14)
C19—H19*C*···*Cg*1^v^	0.995 (19)	2.947 (19)	3.9072 (19)	162.0 (15)

Symmetry codes: (i) *x*−1, *y*+1, *z*; (ii) *x*+1, *y*−1, *z*; (iii) −*x*, −*y*+1, −*z*+1; (iv) −*x*+1, −*y*+1, −*z*+1; (v) *x*, *y*+1, *z*.
